# Arsenic toxicosis in sheep: The first report from Iran

**DOI:** 10.2478/intox-2013-0016

**Published:** 2013-06

**Authors:** Javad Ashrafihelan, Jamileh Salar Amoli, Mehran Alamdari, Tahereh Ali Esfahani, Morteza Mozafari, Ali Reza Nourian, Ali Asghar Bahari

**Affiliations:** 1 Department of Pathobiology, Faculty of Veterinary Medicine, University of Tabriz, Tabriz, Iran; 2 Toxicology Research Center, Faculty of Veterinary Medicine, University of Tehran, Tehran, Iran; 3 Veterinary Organization, Tabriz, Iran; 4 Faculty of Veterinary Medicine, University of Tabriz, Tabriz, Iran; 5 Department of Pathobiology, Faculty of Paraveterinary Medicine, Bu-Ali Sina University, Hamedan, Iran; 6 Department of Clinical Sciences, Faculty of Paraveterinary Medicine, Bu-Ali Sina University, Hamedan, Iran

**Keywords:** arsenic, chronic poisoning, small ruminant, ghopuz, iran

## Abstract

Arsenic contamination of groundwater has been previously reported in Ghopuz, a village located in the Northwest of Iran. Samples were taken from consuming and irrigation water and plants of the region for chemical analysis. A seven-year old ewe, which had lived in and fed a lifelong at the same place, with clinical signs such as weakness, wasting and inappropriate integument was necropsied. Grossly, buccal erosion, stomatitis, cutaneous ulcers and serous atrophy of fat deposits were observed. Rumen contents, wool and several tissue samples were obtained for toxicological and histopathological examinations. Mean arsenic concentration in the spring water, irrigation water and grass/algae were 70.11, 48.74 and 141.85 ppb (µg/kg), respectively. Arsenic levels were 486.73, 247.94, 127.92, 125.97 and 231.24 ppb in wool, skin, rumen contents, liver and kidney, respectively. Microscopic study revealed hyperemia and heavy parasitic infestation of the abomasal wall. Hyperemia and regeneration of renal tubule epithelia were observed in kidneys and hyperkeratosis, suppurative deep dermatitis and paniculitis were found in skin. Periacinar fibrosis and a poorly differentiated cholangiocarcinoma were seen in liver. In pancreas, reduced cell density of islands of Langerhans was noticeable. In the central nervous system, perineuronal and perivascular edema, ischemic changes in gray matter neurons, and microcavitation of white matter were present. Our findings confirmed chronic arsenic toxicosis in small ruminants in this region. It can be concluded that long-term consumption of arsenic contamined water and forage may be associated with chronic arsenic poisoning in domestic animals and human beings, with consequent neoplastic disease and induction of diabetes in this region.

## Introduction

Arsenic can be found almost everywhere in the Earth's crust (Mandal & Suzuki, 2002; Eisler, 1988). This heavy metal exists in organic and inorganic forms and is frequently used in wood preservatives, herbicides, pesticides, insecticides as ectoparasiticide and wormicidal compounds, food supplements, and drugs. Arsenic poisoning has previously been considered as one of the most common causes of toxicity in domestic animals and birds (Bazargani *et al.*, 2007; Doyle & Spaulding, 1978; Jubb *et al.*, 1985; Selby *et al.*, 1977). Water and forage containing high concentrations of arsenic are the main ways through which arsenic enters the animal body, consequently gets into the human food chain, and may cause poisoning in humans and animals. Arsenic remains one of the most important carcinogens and diabetogenics in human (Biswas *et al.*, 2000; Rana *et al.*, 2010; Mukherjee *et al.*, 2004; Eisler, 1988). Nowadays it is, after lead considered the most common toxic heavy metal affecting domestic animals (Bazargani *et al.*, 2007; Doyle & Spaulding, 1978; Selby *et al.*, 1977). Arsenic poisoning may occur as peracute, acute, subacute or chronic form. While sudden death without particular clinical signs may occur in the peracute form of poisoning, acute and subacute forms are associated with severe gastroenteritis. Chronic poisoning causes ill thrift, weakness and inability, milk reduction and abortion (Bazargani *et al.*, 2007; Radostits *et al.*, 2007; Selby *et al.*, 1977). Presence of this metal in water has been reported from different countries such as USA, Canada, Mexico, Argentina, Chile, Poland, Japan, China, Taiwan, Nepal, Vietnam, Bangladesh, India and Iran (Hosseinpourfeizi *et al.*, 2007; Mandal & Suzuki, 2002; Mosaferi *et al.*, 2008; Ng *et al.*, 2003; Wang *et al.*, 2002). Several cases of chronic arsenic poisoning in humans have been reported from Iran (Hosseinpourfeizi *et al.*, 2007; Mosaferi *et al.*, 2008), there is, however, only one animal report from an industrial cattle farm (Bazargani *et al.*, 2007). In the present report, chronic arsenic poisoning in sheep is discussed, based on clinical manifestations, necropsy, histopathological and toxicological findings.

## Materials and methods

### Sampling and necropsy

In May 2011, during investigation from Ghopuz, a village in Hashtrood, East Azerbaijan, Northwest of Iran, samples was collected from spring water, irrigation water and grass of the region. On clinical examination of small ruminants, clinical signs of chronic arsenic poisoning such as weakness, congestive mucous membranes, emaciation and several ulcers in different parts of skin were noticed. The wool and hooves appeared inappropriate, and focal erosive stomatitis was seen. Adult small ruminants, especially 3 years old or more suffered from chronic arsenicosis. A seven-year-old ewe with the above mentioned clinical signs, which had lived in and fed a lifelong at the same place, was slaughtered and standard necropsy was performed. Tissue samples from kidneys, lymph nodes, liver, pancreas, intestines, abomasum, brain, skin and lung were taken for histopathological examinations. Samples were fixed in 10% neutral buffered formalin, processed with standard histological method and stained with Hematoxylin and Eosin (H&E). Samples from wool, skin, rumen content, blood, urine, bile, liver, kidneys, brain, as well as consuming and irrigation water, plants and algae grown near the water sources were submitted to the Toxicology Research Center, Faculty of Veterinary Medicine, University of Tehran.

### Toxicological test method

For arsenic determination, 4 g representative samples were transferred into dishes, was 10 ml of 2 M HCL added and then digested at 80 °C in water bath for 16–24 hr. In the further step the samples were cooled and filtered in a volumetric flask. Then 200 mg/kg Potassium Iodide (KI) and HCL 2M were added. Finally, after warming in 80 °C water bath for 5–8 minutes with 0.5 ml/1 Octanol, the contents of the dishes were diluted in 25 ml volumetric flasks. Finally the levels of arsenic were determined by 906 GBC Hydride Generation spectrophotometer with limit of detection (LOD) 0.002 ppm.

## Results

### Necropsy findings

**Figure 1:**
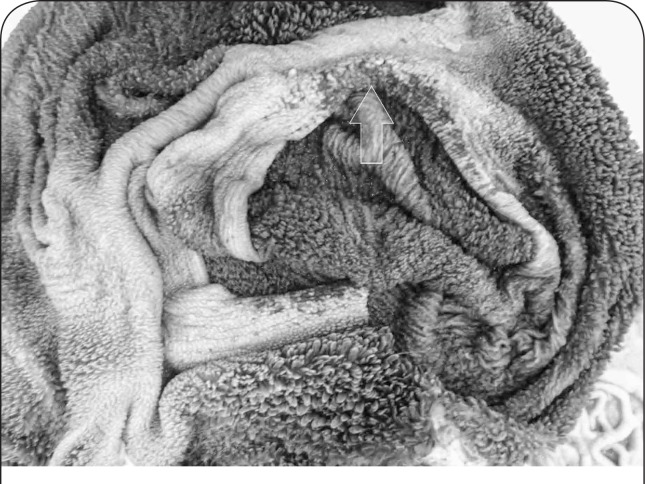
Chronic arsenicosis in a ewe: rumen mucosa is dark and leathery with erosions on pillar surface (arrow).

**Figure 2:**
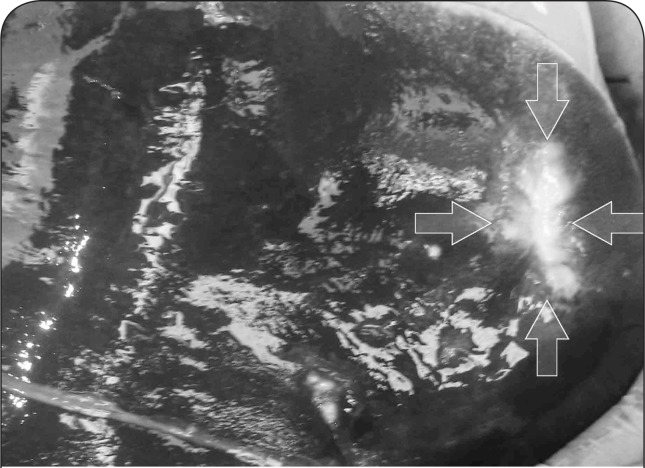
Chronic arsenicosis in a ewe: a solid whitish neoplastic mass, approximately 3.5 centimeters in diameter is seen at right side of parietal surface of liver (arrows).

**Figure 3:**
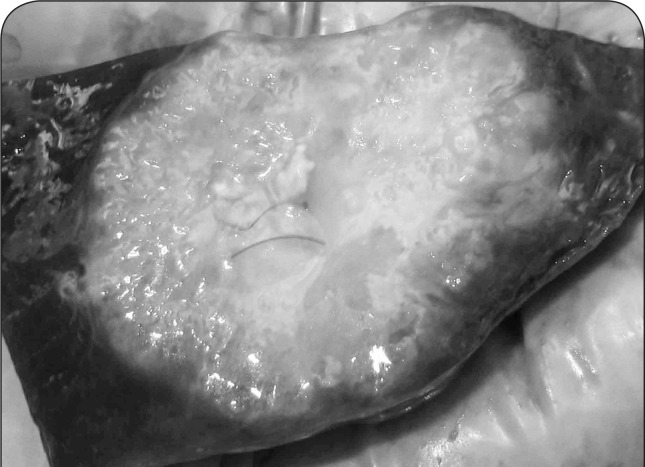
Cut surface of the neoplastic mass is presented in Figure 2. Grossly, the mass was solid, white in color, firm in consistency with relatively homogenous cut surface.

The carcass was cachectic and serous atrophy of fat appeared in subcutaneous, subepicardial and visceral fat deposits. Rumen mucosa was dark and leathery with relatively extensive erosions at thr surface of rumenal pillars (Figure 1). There was extensive hyperplasia in the abomasum wall. Serosae were hyperemic and thickening of the jejunum wall was obvious. There were some degrees of degenerative changes and edema in mesenteric lymph nodes. Parasitic infection, fatty change foci associated with 3 solid round whitish masses, approximately 1, 2 and 3.5 centimeters in diameter were found in the parietal surface of liver (Figures 2 and 3). In lung, adhesion, pseudotuberculosis (caseous lymphadenitis) lesions, emphysematous and parasitic foci were observed. Kidneys were pale, with serous atrophy of perirenal and renal fat.

### Toxicological findings

Several samples from consuming water of Looleh-bolagh spring, irrigation water, grass and algae grown near these sources, as well as wool, skin, rumen contents, blood, urine, bile, kidney, liver and brain of the sheep were analyzed by Hydride-Generation Atomic Absorption Spectroscopy (HG-AAS). Arsenic concentrations in Looleh-bolagh spring water, irrigation water and grass/algae were 70.11, 48.74 and 141.85 ppb (µg/kg), respectively. Arsenic levels were 486.73, 247.94, 127.93, 125.97, 231.24 and 110.40 ppb in samples from wool, skin, rumen contents, liver, kidney and urine, respectively. The arsenic levels in brain, bile and blood were less than 20 ppb (Table 1).

**Table 1:** Arsenic concentrations in samples from material and tissue samples.

Sample	Arsenic concentration (ppb)
Looleh-bolagh spring water	70.11
Irrigation water	48.74
Grass/algae	141.85
Wool	486.73
Skin	247.94
Rumen contents	127.93
Liver	125.97
Kidney	231.24
Urine	110.40
Brain	<20
Bile	<20
Blood	<20

### Histopathological findings

**Figure 4:**
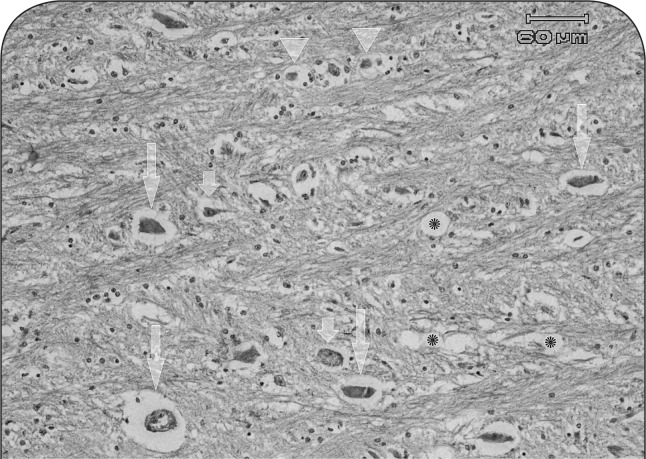
A microscopic view of medulla oblongata in a ewe affected with chronic arsenicosis: perivascular edema (long arrows), ischemic change of large neurons (short arrows), satellitosis (arrow heads), and vacuolation of neuropil (stars) are noticed (H&E, 200×).

Histopathological examinations revealed mild degenerative changes in the central nervous system. Congestion, diffuse gliosis and satellitosis in gray matter with ischemic changes and single cell necrosis in large pyramid neurons of the third layer were observed. In the white matter, perineuronal and perivascular edema and vacuolation were noticed. Congestion, perineuronal and perivascular edema, microcavitation and status spongiosis were seen in cerebellum, medulla oblongata and some brain nuclei (Figure 4).

Acanthosis, mild hyperkeratosis and parakeratosis and hydropic degeneration of squamous cells was observed in rumen epithelia. The abomasum was severely hyperemic and edematous and heavily infested with parasites. Abomasal glands were penetrated with parasites so that abomasal mucosa was severely hyperplastic and glands were distended and filled. Mononuclear gastritis with predominance of lymphocytes was found. Lymphocytic enteritis was seen. Kidneys were congested and hypercellularity of glomeruli and regeneration of renal tubular epithelia was noticed.

Severe hyperkeratosis and pustules formation were seen in the skin. Hyperemia and accumulation of fibrin and pus in subcutis (suppurative paniculitis) also occurred.

**Figure 5:**
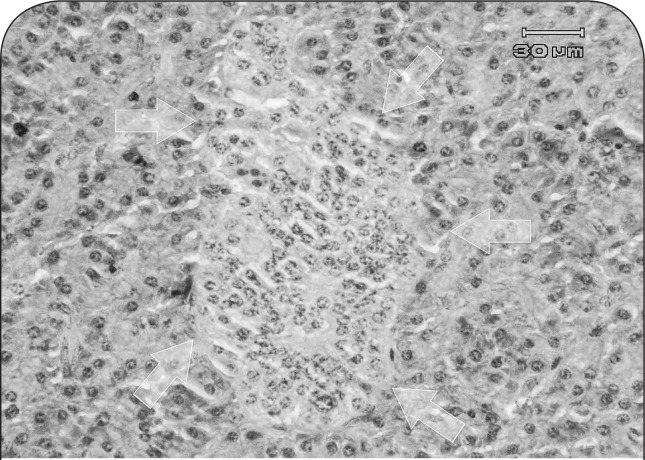
Microscopic view of endocrine pancreas in a ewe affected with chronic arsenicosis: a relatively normal islet of Langerhans (arrows) is observed (H&E, 400×).

**Figure 6:**
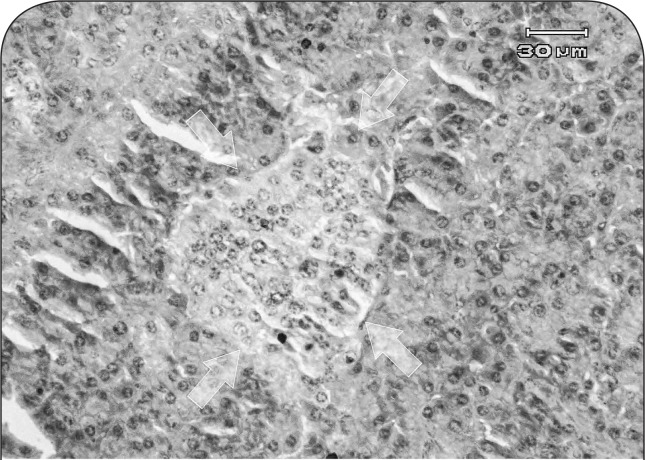
Histopathological section of endocrine pancreas in a ewe affected with chronic arsenicosis: hypocellularity and evidence of pyknosis and regeneration of nuclei are seen in an affected islet of Langerhans (arrows) (H&E, 400×).

Regeneration of cells was noticed in the endocrine pancreas, the nuclei were large and hyperchromatic. Additionally, density of cells in islets of Langerhans was reduced in some areas or no cell was present in some islets (Figures 5 and 6).

**Figure 7:**
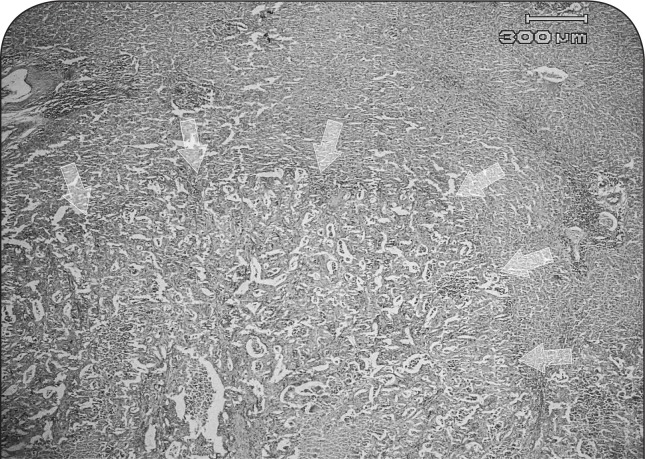
Microscopic view of tumor in liver of a ewe affected with chronic arsenicosis: the mass (arrows) consists of neoplastic cells arranged in acinar-like structures and diagnosed as cholangiocarcinoma (H&E, 40×).

**Figure 8:**
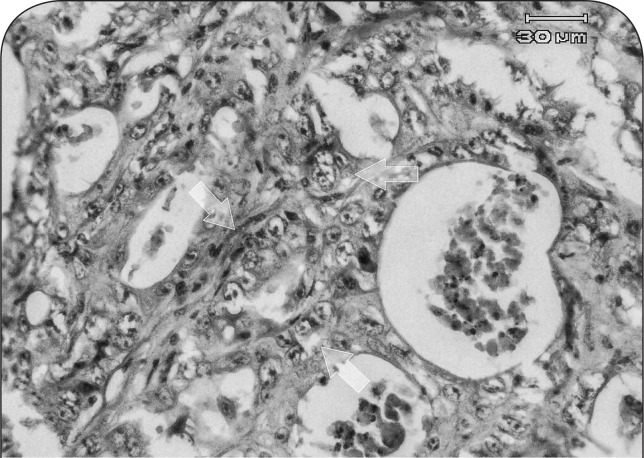
Microscopic section of cholangiocarcinoma in liver of a ewe affected with chronic arsenicosis: neoplastic cells are arranged in acinar-like structures and tend to form lumen as bile ducts (arrows) (H&E, 400×).

In liver, mild fatty changes, focal necrosis, mononuclear cholangiohepatitis, proliferation of connective tissue in periacinar regions and hyperplasia of bile ducts in the portal areas were noticeable. The hepatic masses were diagnosed as poorly differentiated cholangiocarcinoma (Figure 7). Neoplastic cells were arranged in acinar structures and tended to form a lumen as bile ducts (Figure 8) and exhibited severe pleomorphism, karyomegaly and bizarre shapes. The nuclei of neoplastic cells were hyperchromatic and variable in both size and shape. Nuclear to cytoplasmic ratio (N/C), which in normal hepatocyte is ^1^/_6_ to ^1^/_4_ was markedly increased to ^1^/_1_ and even ^2^/_1_ in neoplastic cells, and the mitotic index was 5 to 6 mitoses in a microscopic field of 400 magnification.

## Discussion

Arsenic occurs in organic and inorganic forms, each of which includes trivalent and pentavalent forms; however, the former is much more toxic than the latter. The toxicity is different, based on the exposed amount and on the solubility of these compounds. Soluble salts of trivalent and pentavalent inorganic arsenic are highly toxic to humans and animals (Bazargani *et al.*, 2007; Biswas *et al.*, 2000; Gupta, 2007; Jubb *et al.*, 1985; Plumlee, 2004; Wang *et al.*, 2002; Rana *et al.*, 2010).

Accidental access of animals to arsenical pesticides and herbicides, contamination of pastures, forage and drinking water with fume and dust produced by smelting plants, licking wood treated with arsenic preservatives, and dipping animals in arsenic contained pesticide solutions for ectoparasite treatment are common ways of exposing domestic animals to arsenic.

In addition, an important mode of human and animal exposure to arsenic is consuming groundwater with high levels of arsenic. This is a global concern since arsenic is thought to be carcinogenic (Banejad & Olyaie, 2011; Bazargani *et al.*, 2007; Biswas *et al.*, 2000; 4, 7, Gupta, 2007; Hosseinpourfeizi *et al.*, 2007; Jubb *et al.*, 1985; Plumlee, 2004; Mandal & Suzuki, 2002). The presence of arsenic in water and chronic ailment of the human population in the Hashtrood region has been reported by researchers from Tabriz University of Medical Sciences in 2007 and 2009 (Hosseinpourfeizi *et al.*, 2007; Mosaferi *et al.*, 2008). In the present study, the source of poisoning was found to be contamination of underground water of the region with arsenic. The arsenic level of Looleh-bolagh spring water, which is only used as source of drinking water for domestic animals and irrigation of crop, was 70.11 ppb (ng/ml), which is higher than the former and present Iranian national standard and World Health Organization guideline of 50 ng/ml, 10 ng/ml and 10 ng/ml, respectively (World Health Organization, 2004; Hosseinpourfeizi *et al.*, 2007; Mosaferi *et al.*, 2008; Institute of Standards and Industrial Research of Iran, 2010).

The mechanism of action of arsenic is believed to be the inhibition of enzymes and structural proteins through binding to sulfhydryl groups of proteins; therefore organs rich in oxidative systems such as liver, kidneys and skin are more susceptible to toxic effect of this element (Gupta, 2007; Jubb *et al.*, 1985; Plumlee, 2004; Radostits *et al.*, 2007; Rana *et al.*, 2010; Tchounwou *et al.*, 2003). In addition, arsenicals are known as tumorigenic compounds, the mechanism of which is not completely established., They may act through inhibition of DNA repairing enzymes, and/or the capability of arsenic as analogues of phosphate may be associated with its tumorogenicity. Moreover, it was found that arsenic can induce some growth factors and hence cause cellular proliferation and eventually cancer (Chou *et al.*, 2000; Tchounwou *et al.*, 2003; Wang *et al.*, 2002). All research data on carcinogenicity of arsenic are related to human and animal models, such as the mouse (Ng *et al.*, 2003). In the authors’ knowledge, no data on tumors due to arsenic poisoning in ruminants have been published. Although arsenicals have been classified as carcinogens in people, this has not been the case in animals (Gupta, 2007). Nevertheless, since the ability of arsenic in hepatic tumor induction has been proven (Centeno *et al.*, 2002; Chen *et al.*, 2004), the cholangiocarcinoma found in this study may be attributable to long-time exposure to sublethal amounts of arsenic.

The usual clinical signs of chronic arsenicosis, including weight loss, weakness, languor, ill thrift, dry and alopecic coat that readily comes off, focal skin lesions, congested mucous membranes and stomatitis (Radostits *et al.*, 2007), were all seen in the sheep studied.

One of the primary target organs for chronic arsenic poisoning is the nervous system, in which mild edema of white matter of brain and spinal cord as well as shrinkage and permutation of several neurons of medulla oblongata occur (Plumlee, 2004; Radostits *et al.*, 2007). Hepatic necrosis and hemorrhagic enteritis are some other important lesions of arsenic poisoning (Bazargani *et al.*, 2007; Biswas *et al.*, 2000; Jubb *et al.*, 1985; Selby *et al.*, 1977). Abomasal and intestinal hyperemia as well as enteritis were seen in this study; yet severe parasitic infestation of abomasum may have partially been responsible for leanness and weakness of the animal. In liver, necrotic foci along with cholangiohepatitis were detected. Kidneys were congested and evidence of regeneration of renal tubular epithelial cells was obvious. Furthermore, congestion and diffuse gliosis in the CNS along with ischemia and perineuronal and perivascular edema in cerebrum, cerebellum and medulla oblongata were found. In this study, lesions of the central nervous system were mild. In our opinion, this was due the to low concentration of As in this organ. In the study of Biswas *et al.* on goat chronic arsenicosis, the brain retained the least residues (5.18±0.04 µg/g) and presented only mild congestion, edema and degenerative lesions.

Cellular density of the endocrine pancreas was decreased. This may have been associated with arsenic toxicity, however, no report was found in the literature regarding this issue in ruminants and was reported only in the rabbit (Mukherjee *et al.*, 2004).

Chronic arsenicosis occurs in particular organs such as liver, kidneys, epidermis, spleen and lungs duo to accumulation of arsenic (Radostits *et al.*, 2007); however, since chronic poisoning is not common in animals (Plumlee, 2004), there is no available data on arsenic levels in sheep organs. Even in cases of acute and subacute poisoning, due to the wide variety of reported levels, interpretation of toxicological results should be carried out cautiously (Bazargani *et al.*, 2007).

From the toxicological point of view, the liver is the best organ for investigating arsenic, particularly in chronic cases in the kidney, the level of arsenic in kidney may be higher than in the liver (Radostits *et al.*, 2007), which was the case in the present study. There is little agreement in the literature regarding normal concentrations of arsenic in different organs of sheep. Its level has been reported to be within the range of 50–150 ppb in the liver and 30–150 ppb in the kidneys (Doyle & Spaulding, 1978). In this study, the arsenic concentrations in liver and kidneys were 125.97 and 231.24 ppb, respectively. These concentrations are higher than normal level and thus may be hazardous for consumers.

Considering the clinical signs, gross and histopathological findings and toxicological results, chronic arsenic poisoning in the animal studied is likely to be established.

In addition, since water was the main source of this poisoning, determination of the arsenic concentration in different sources of water and plants in the region is necessary in order to reduce the undesirable consequences of arsenic uptake by domestic animals and direct and indirect entry of arsenic in the human food chain in the region.
